# Efficacy of a Culture-Specific Dancing Programme to Meet Current Physical Activity Recommendations in Postmenopausal Women

**DOI:** 10.3390/ijerph17165709

**Published:** 2020-08-07

**Authors:** Jennifer Hargan, Emilie Combet, Paul Dougal, Mhairi McGowan, Mary Ann Lumsden, Dalia Malkova

**Affiliations:** School of Medicine, Dentistry and Nursing, College of Medicine, Veterinary and Life Sciences, University of Glasgow, New Lister Building, Glasgow Royal Infirmary, Glasgow G31 2ER, UK; jennifer.hargan91@gmail.com (J.H.); Emilie.CombetAspray@glasgow.ac.uk (E.C.); paul.dougall@sky.com (P.D.); mhairi_mcgowan@msn.com (M.M.); Maryann.Lumsden@glasgow.ac.uk (M.A.L.)

**Keywords:** postmenopausal women, physical activity recommendations, cardiorespiratory fitness, dancing

## Abstract

This study investigated the efficacy of participation in culture-specific dancing to meet current physical activity recommendations and increase cardio-respiratory fitness in postmenopausal women. Sedentary postmenopausal women (n = 24), aged 63 ± 8 years and with BMI of 28 ± 3 kg/m^2^ completed a 4-week Scottish dancing study. The dancing sessions of approximately 75 min were performed twice a week and each session was based on five Scottish dances performed in 3 sets. Heart rate (HR) measurements were obtained during all dances to evaluate whether the intervention achieves the criteria of moderate to vigorous aerobic exercise intensity. Body composition, waist circumference, and HR during Chester Step test were measured before and after dancing intervention. HR achieved during individual dances ranged from 64 ± 5% to 80 ± 5% of HRmax and the mean HR of the five dances corresponded to 72 ± 7% of HRmax. Post-intervention mean HR was lower throughout Level 2 (Pre, 112 ± 13 bpm; Post, 106 ± 13 bpm; *p* = 0.005) and Level 3 (Pre, 122 ± 14 bpm; Post, 115 ± 14 bpm; *p* = 0.006) of the Chester test compared with baseline values. The intervention had no impact on body weight or body fat but reduced waist circumference (Pre, 94 ± 8 cm; Post, 91 ± 9 cm; *p* = 0.006). Thus, traditional Scottish dancing should be advocated to sedentary postmenopausal women, emphasising its potential in meeting current physical activity recommendations in relation of weekly duration and exercise intensity and improving cardiorespiratory fitness.

## 1. Introduction

Despite the estimation that physical inactivity is responsible for 1 in 10 deaths globally [1], physical activity (PA) levels continue to decline. Less than one-third of the population in Europe and the US achieve the PA guidelines as currently advised [2]. In the US, PA guidelines recommend that for substantial health benefits, healthy adults aged 18−65 undertake either a minimum of 30 min of moderate PA on 5 or more days of the week, or 20 min of vigorous aerobic activity on three days of the week, achieving a weekly total energy expenditure of ≥500–1000 MET-minutes [3]. The major recent developments, such as the 2018 Physical Activity Guidelines for Americans and WHO Global Action Plan for Physical Activity, recommend that adults should do at least 150 to 300 min a week of moderate intensity, or 75 to 150 min a week of vigorous intensity aerobic exercise, or an equivalent combination of moderate and vigorous aerobic activity [4,5]. The current UK guidelines are very similar, stating that each week, adults should accumulate at least 150 min of moderate intensity activity, or 75 min of vigorous intensity activity, or even shorter durations of very vigorous intensity activity, or a combination of moderate, vigorous and very vigorous intensity activity [6]. These recent PA guidelines imply that moderate to vigorous intensity exercise PA, by being key component of PA, should be combined with muscle strengthening and balance improving components of PA and that the guidelines for adults are relevant to healthy older adults [4,5,6].

The evidence based on data from countries representing 96% of the world’s population suggest that, in almost every country, women are less physically active than men and that a substantial gender gap has changed little over time [2,7,8,9]. In women in high-income countries, the prevalence of insufficient PA (defined as not achieving, weekly, at least 150 min of moderate-intensity or 75 min of vigorous intensity PA, or any equivalent combination of the two) is particularly high (42%, 95% confidence interval from 39% to 45%) [7]. Furthermore, PA levels in women decline progressively with the aging process; a sedentary lifestyle is very common in older women, including those who are at the early stages of post-menopause [10]. The decline in PA level, together with reduction in endogenous oestrogen secretion, is an underlying reason for postmenopausal overweight and obesity and several health complications, including enhanced risk of cardiovascular diseases, a major course of death in elderly women [11]. In addition, low PA levels in postmenopausal women contribute to age-induced reduction in cardiorespiratory fitness, which has been extensively associated with enhanced risk of cardiovascular disorders such as myocardial infarction, stroke, and peripheral vascular disease [12,13]. Thus, identifying strategies and type of exercise programmes allowing postmenopausal women to achieve current PA recommendations is of great importance.

Despite evidence that participation in exercise improves markers of cardiovascular health in postmenopausal women [14,15,16], numerous factors deter women in this age group from engaging in PA. Dislike of exercise and lack of interest and enjoyment are reported amongst major barriers [8]. Thus, most exercise interventions in postmenopausal women have low adherence and high dropout rates [17], implying that traditional forms of exercise may not be successful in promoting PA in this group. At the same time, there is evidence to suggest that dance-based exercise might make exercise more interesting and attractive to older women [18] and be as effective as other types of exercise to enhance cardiorespiratory fitness and yield meaningful health benefits [19,20]. Nevertheless, studies evaluating impact of dance as a form of exercise on cardiorespiratory fitness in this group are scarce. In addition, only a few dance interventions considered traditional dances, reflecting the roots and identities of specific regions.

This study aimed to investigate the efficacy of participation in traditional Scottish dancing to meet current PA recommendations in relation to weekly duration and intensity of exercise sessions in postmenopausal women. It additionally, it investigated whether participation in traditional Scottish dancing can modify body fatness and central obesity measured by waist circumference and increase cardio-respiratory fitness, a robust predictor of cardiovascular risk and other adverse health outcomes. Our decision to investigate efficacy of Scottish traditional dancing is related to the evidence that PA levels are concern in Scotland, where only 12% of women aged over 65 achieve the current guidelines [10].

## 2. Methods

### 2.1. Participants

The participants in this study were healthy postmenopausal women, aged 50–80 years. Participants were previously sedentary, i.e., performed little or no organized PA and were spending most waking hours sitting. Participation in PA was estimated from recall diaries reflecting organized and non-organized activities (walking, cycling for transportation) over two months prior to participation in the dancing programme. Prospective participants were recruited via online and leaflet advertisement, targeting women with a BMI in the category for overweight (BMI > 25kg/m^2^) or obesity (BMI > 30 kg/m^2^), and briefed individually by an investigator who provided them with a participant information sheet outlining the requirements of the study. Participants were required to read and consent to the risk assessments associated with the study, and to complete a PA readiness questionnaire (PAR-Q) and a health-screening questionnaire prior to commencing the study. Women were invited to participate if answers to all PAR-Q question were negative. Participants were required to be non-smokers, with stable body weight for two months prior to the study enrolment, free of nutritional supplementation or following any specific diet. Women were not eligible if they had suffered from myocardial infarction, heart failure, or active angina in the previous 6 months or had blood pressure greater than 160/90mmHg. Participants with chronic illness, eating disorders and history of gastrointestinal operations were also excluded. The Ethics Committee of the College of Medical Veterinary and Life Sciences of the University of Glasgow approved the study (Project No 2012051). Recruitment and data collection started in August 2012 and study was completed in March 2013. Thirty-nine women were initially recruited (21 online, 16 via poster advertisement and 2 by word of mouth). Although all women met the inclusion criteria, 7 did not participate due to unsuitability of class times. Therefore, 32 participants started the intervention.

### 2.2. Study Design

This was an open-label, single-arm study. Women participated in a closely supervised Scottish dancing programme (2 × 75 min of dancing sessions per week, over 4 weeks), designed to achieve current PA recommendations in relation to weekly duration of exercise [4,5,6]. The dancing programme was specifically designed for this study and was tailored to achieve moderate to vigorous intensity aerobic exercise, another feature of PA guidelines [4,5,6], and enhance cardio-respiratory fitness. Regular attendance was encouraged, and telephone calls were made to determine reasons for non-attendance. Heart rate responses during five traditional Scottish dances of varying intensity were measured to calculate %HRmax to be used for determining the exercise intensity zones according to criteria established by the American College of Sport Medicine [21]. Body weight, body composition and cardio-respiratory fitness were measured at baseline, and 12–16 h after the final exercise session.

### 2.3. Anthropometry

Height was measured via a Stadiometer model (SECA 213, Leicester, UK). Waist circumference was obtained at the end of normal expiration at the narrowest point of the abdomen using a standard waist measuring tape. The anthropometric measurements were taken twice on both occasions (before and after intervention), according to International Society for the Advancement of Kinanthropometry (ISAK) standards [22]. Bioelectrical impedance analysis (*TANITA TBF−310, Cranela, UK*) was used to measure weight, BMI (weight (kg)/ height (m)^2^), fat mass and fat-free mass. For all measurements, participants required to be fasted, wearing minimal clothing and no shoes.

### 2.4. Chester Step Test

Cardio-respiratory fitness was assessed via a modified version of the Chester step test [23]. The test consisted of five incremental stages using a 20-cm high step (Reebok, Taiwan) during which a pre-recorded metronomic CD was employed to set the pace of stepping. The test was initiated at a pace of 15 steps per minute and as the test proceeded the pace was increased by 5 steps per minute. Each level lasted two minutes and was followed by a two-minute break. The maximum pace corresponded to an intensity of 35 steps per minute. Heart rate (Polar RS400 or S610i, Polar Electro Oy, Kempele, Finland) and rating of perceived exertion (RPE) [24] were recorded during the final seconds of Level 1, 2 and 3, and for each participant mean of HR and RPE were calculated as average of values recorded during the last few seconds of Level 1, 2 and 3 of Chester Step test. The test was terminated when participants reached 80% of their maximal HR (HR_max_), with HR_max_ being calculated as 220 −age. If 80% of maximal HR was reached during a level, yet participants appeared to be in no great physical strain, the level was completed before terminating the test. During pre- and post- training intervention, Chester step tests were conducted in the morning, at approximately the same room temperature. Prior to the first Chester test, participants were advised to drink some water. This amount of water was recorded and replicated during the second test.

### 2.5. Intervention

The dancing programme was designed and facilitated by a Scottish dance expert. The 4-week dancing intervention required participants to attend 2 sessions per week, each lasting approximately 90 min including breaks. All sessions consisted of three sets and each set was comprised of 5 traditional Scottish dances lasting 4–5 min, with a 1–2 min break permitted between dances. A break of 2–4 min was allowed between sets. Thus, active dancing during each of the sessions lasted for approximately 75 min. Each set was designed to vary exercise intensity in the following pattern: low (St. Bernards Waltz), moderate (Gay Gordons), vigorous (Canadian Barn Dance), moderate (Gay Gordons), and vigorous (Strip the Willow). As the intervention was specifically tailored for the current participants, techniques which were considered too strenuous, such as spins, were replaced with easier stepping movements. HR and RPE were measured at the very end of five dances of the second and third set of the sessions conducted in week 3 and week 4, when participants were familiar with the routines. Towards of the end of each dance, randomly selected participants were reminded and helped to take HR and RPE measurements and asked to report these measurements to the researcher. The obtained data were used to calculate group mean and SD values. Dancing sessions were performed in the late afternoon, with dancing session starting at approximately 18:00. Each exercise session was supervised by the researcher.

### 2.6. Statistical Analysis and Calculations

The data collected were tested for normality via the Anderson–Darling test. Since all variables were normally distributed, the differences between pre- and post-intervention values were determined via paired *t*-tests. Statistical significance was considered when *p* < 0.05. Categorical data were analysed using Fishers Exact test. Statistical analysis was conducted by Minitab (version18, Minitab Inc., State College, PA, USA). Adherence to the dancing programme was calculated from the participation logs by using the following formula: (the number of sessions completed/ the number of sessions prescribed) × 100%.

## 3. Results

The dancing programme was completed by 28 women and the study achieved an adherence rate of 89 ± 13%. Two participants failed to attend the post-intervention assessment session and 2 did not supply full data. Thus, the results are presented on 24 women (75% of participants who enrolled the dancing programme). Baseline characteristics of the 24 women are presented in Table 1.

The intensity of each of the five dances used in the intervention was estimated from HR recordings conducted on a subset of participants (n = 14). A total of 815 HR and 818 RPE measurements were recorded throughout the intervention. Table 2 presents mean RPE and mean HR, calculated from averaged individual RPE and HR values recorded during the second and third set of the week 3 and 4, HR as percentage of predicted individual maximal heart rate (%HR_max_) for each dance and the corresponding exercise intensity according to criteria established by the American College of Sport Medicine for [21]. The Canadian Barn Dance was the most intense form of exercise (80 ± 5% HR_max_), followed by intermediate intensity dances; Strip the Willow (75 ± 5% HR_max_), Virginia Reel (73 ± 5% HR_max_) and the Gay Gordons (70 ± 3% HR_max_), and St. Bernards Waltz was the least intensive dance (64 ± 5% HR_max_). The average HR throughout the dancing sessions was 113 ± 11 bpm, corresponding to 72 ± 7% HR_max_.

Pre- and post-intervention measures of body weight, body composition, BMI, and waist circumference are displayed in Table 1. No significant changes in body weight, BMI or percentage body fat were detected following the intervention. However, there was a significant reduction in waist circumference of 2.3 ± 3.6 cm (*p* = 0.006).

**Figure 1:**
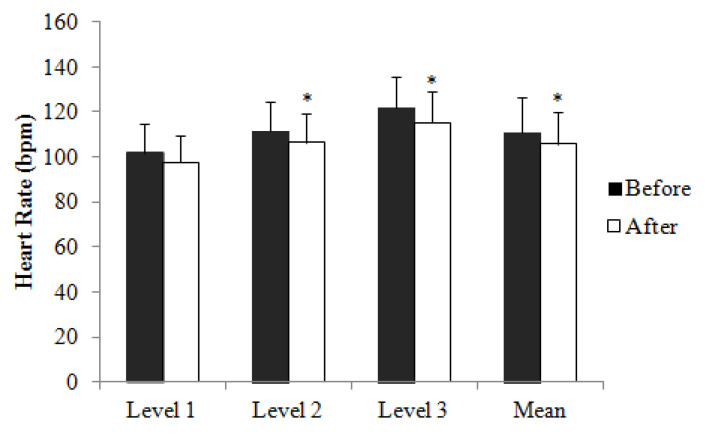
Heart rate (HR) recorded during the final seconds of Level 1, 2 and 3, and Mean HR calculated from averaged individual HR values recorded during the final seconds of Level 1, 2 and 3 of Chester Step test conducted before and after dancing intervention. Values are mean ± SD. * Significant difference (*p* < 0.05, paired *t*-test) from before.

Table 3 shows the number of participants who completed each level of the Chester Step Test pre- and post-intervention. Three participants were unable to perform the step test due to knee problems. Therefore, data from 21 women were assessed regarding change in cardio-respiratory fitness. HR and RPE were compared for those who completed Level 1, 2 and 3 pre- and post-intervention. Table 4 and Figure 1 shows pre- and post-intervention HR recorded during the final seconds of Level 1, 2 and 3, and mean HR calculated from averaged individual HR values recorded during the last few seconds of Level 1, 2 and 3 of the Chester Step test. No difference was noted between measurements at Level 1 while HR was significantly lower post-intervention during Level 2 (Pre, 112 ± 13 bpm; Post, 106 ± 13 bpm; *p* = 0.005) and Level 3 (Pre, 122 ± 14 bpm; Post, 115 ± 14 bpm; *p* = 0.006). Mean HR of the first three levels of step test was significantly lower post-intervention (Pre, 111 ± 15 bpm; Post, 106 ± 14 bpm; *p* < 0.001). There was no statistical difference in RPE throughout the first three levels of the Chester Step test.

## 4. Discussion

The current study demonstrated that, for sedentary postmenopausal women, participation in traditional Scottish dancing provides a novel strategy for meeting current PA recommendations, in relation to the weekly duration and intensity of aerobic exercise [3,4,5,6]. We also found that postmenopausal women participating in Scottish dance-based exercise increased cardio-respiratory fitness, an independent predictor of cardiovascular risk, and cardiovascular and total mortality [25,26,27]. Thus, taking into consideration these outcomes and finding that the adherence rate achieved in our study was high and at a level which is required for exercise intervention to be satisfactory in providing health benefits [28], our study suggests that traditional Scottish dancing could be advocated to sedentary postmenopausal women.

The 4-week Scottish dancing programme required participants to attend two weakly 90-min sessions, with the active dancing component consisting of 75 min. The dances chosen for the programme were specifically tailored to vary the intensity of the exercise sessions, ranging from light- to-moderate to vigorous intensity. According to the ACSM [21], the HR measurements indicated that during three dances (Gay Gordons, Virginia Reel, Strip the Willow) women exercised at moderate intensity and that light-to-moderate and vigorous exercise intensities were achieved during St. Bernard’s Waltz and Canadian Barn dances, respectively [21]. On average, study participants exercised at 72 ± 7% HR_max_ which represents moderate intensity exercise according to the ACSM [21]. Thus, our data suggest that the Scottish dancing programme allowed postmenopausal women to meet criteria of current PA recommendations in relation to both weekly exercise duration and intensity [4,5,6]. We note that exercise sessions were very well attended, with an adherence rate of 89 ± 13%, suggesting that this mode of exercise is acceptable to postmenopausal women and should be considered when devising health policy aimed at enhancing PA levels. Since older people’s adherence rates to exercise interventions are multifactorial and depend not only to the type of the programmes but also on person-level factors [29], the high adherence rate found in this study could be a reflection of participants’ relatively high socioeconomic status, good physical and psychological health, and cognitive ability. However, the impact of these macrosocial variables was not investigated in this study.

As low cardiorespiratory fitness is an independent predictor of cardiovascular risk, and cardiovascular and total mortality [25,26,27], optimising cardio-respiratory fitness is of considerable importance. We found that compared to the pre-intervention test, more participants were able to complete Levels 3, 4 and 5 of the Chester Step test and that meant that HR, calculated from averaged individual HR values recorded during the last few seconds of Level 1, 2 and 3 of the Chester Step test, was lower following the intervention, suggesting that cardiorespiratory fitness was improved in response to the dancing programme. Thus, our findings contribute to the evidence available from recent systematic review and meta-analyses, suggesting that dance interventions increase cardiorespiratory fitness in the elderly [19]. Although the reduction in mean HR during the Chester Step test following the intervention was small (5 ± 9 bpm), effects are likely to be dose dependent [30]. Therefore, if the duration of intervention could have been extended, the improvements recorded would be expected to be amplified. The increase in cardiorespiratory fitness induced by Scottish and other dancing programmes [18] is of great importance since cardio-respiratory fitness appears to have more of an influence on health-protective effects than PA levels. [30].

It was unsurprising that body weight and body fatness measurements were unchanged by the dancing intervention. Alternative dancing and exercise studies in older women have also failed to find a significant reduction in body fat and BMI after aerobic training, [31,32]. We note that the Scottish dancing programme achieved 150 min of moderate to vigorous intensity PA per week, which has been recommended to improve health [4,5,6]. It is well established that body weight loss can be expected only when 200–300 min per week of moderate intensity exercise is accumulated and, for clinically significant body weight lost, weekly duration should exceed 250 min [33]. Thus, lack of changes in body weight and body fatness may be due to the dancing programme being not long enough and thus accumulating energy expenditure to a level equivalent to only small and, most likely, hard to measure body weight loss. Energy expenditure of individual dancing sessions and thus whole 4-week intervention was not measured in this study. However, based on our previous 4-week exercise intervention, during which participants exercised at intensity corresponding to 70–75% HRmax [34] and thus at intensity equivalent to the averaged intensity of the dancing programme, we assume that during 4-week dancing programme our participants expended approximately 3500–4000 kcal. Since for 1kg body mass reduction, creation of a 7400- or 7700-kcal deficit is needed, the energy expended during dancing intervention would be equivalent to approximately 0.46–0.53 kg of body weight loss. Thus, in the absence of caloric restriction, the body weight loss with of short duration exercise is low. In addition, body weight reduction during exercise programmes is compromised by behavioural changes, which include compensatory increase in energy intake [35] and reduction in PA and thus energy expenditure outside exercise training sessions [36]. Nevertheless, waist circumference, a powerful predictor of CVD risk status [37], decreased significantly following the intervention. Thus, the pragmatic minimum target of 150 min of moderate intensity dancing per week brings health benefits beyond significant reduction in body mass and body fat.

This study has limitations. Admittedly, cardiorespiratory fitness is most accurately measured via a maximal oxygen uptake test, which measures the rate of oxygen consumption during either exercising on a treadmill or a bike at an intensity that increases every few minutes until exhaustion and is designed to achieve a maximal effort [21]. However, such measurements involve strenuous exercise and thus were deemed unsuitable for the participants of the current study. In addition, expired air collection and analyses to obtain oxygen consumption were not possible in this study. Nevertheless, studies investigating the prediction of aerobic capacity via the Chester Step test in comparison to maximal oxygen uptake tests imply that predictions of aerobic power from the Chester Step test are not significantly different from those measured via a maximal oxygen uptake test and thus provide valuable estimation of cardio-respiratory capacity [38]. In addition, this study has no control group and our data did not compare the impact of traditional dancing with the impact of other forms of exercise in improving the cardiorespiratory fitness. Predictors of adherence to the dancing programme should also be investigated in future as well as longer term adherence to the different types of exercise.

## 5. Conclusions

The current study highlights that suggests that the pragmatic minimum target of 150 min a week of well-structured, culture-specific (Scottish) dancing programme can not only sufficiently meet PA guidelines, but additionally improve cardiorespiratory fitness and achieved significant reduction in waist circumference, whilst enjoying a low drop-out rate and high rate of adherence in postmenopausal women. Data of this study, however, cannot be used to suggest that participation in a Scottish dancing programme favourably impacts markers of cardiovascular risk and other aspects of health in older women. Thus, this preliminary study indicates that further randomised controlled trials investigating the longer effects of traditional dancing on cardiorespiratory fitness and cardiovascular risk factors in more variable group of postmenopausal are warranted.

## Figures and Tables

**Table 1:** Anthropometric and body composition measurements pre- and post-dancing intervention. Values are presented as mean ± SD, n = 24.

Variable	Pre	Post	Difference	95%CI	*p*-Value
Body Weight (kg)	70.7 ± 7.4	70.5 ± 7.2	0.19	−0.24, 0.62	0.371
BMI (kg/m^2^)	27.8 ± 2.8	27.7 ± 2.7	0.07	−0.10, 0.24	0.383
WC (cm)	93.6 ± 8.5	91.4 ± 8.7	−2.25	−3.38, −0.71	0.006
Body Fat (%)	38.9 ± 3.5	38.7 ± 3.9	0.27	−0.19, 074	0.320

Abbreviations: BMI, Body Mass Index; WC, waist circumference; CI, confidence interval for the difference between means.

**Table 2:** Mean ratings of perceived exertion (RPE) and mean heart rate (HR) calculated from averaged individual RPE and HR values recorded during dances of the second and third set of weeks 3 and 4 and HR as percentage of predicted individual maximal heart rate (% HR_max_). Values are Mean ± SD, n = 14.

Dance	RPE	HR (bpm)	% HR_max_	Intensity *
St. Bernard’s Waltz	1 ± 1	100 ± 7	64 ± 5	Light-Moderate
Gay Gordons	2 ± 1	110 ± 5	70 ± 3	Moderate
Virginia Reel	3 ± 1	115 ± 5	73 ± 5	Moderate
Strip the Willow	3 ± 1	117 ± 5	75 ± 5	Moderate
Canadian Barn	3 ± 1	124 ± 5	80 ± 5	Vigorous
Mean	2 ± 1	113 ± 11	72 ± 7	Moderate

* Classification of exercise intensity is based on criteria of American College of Sports Medicine [21].

**Table 3:** The number of participants who completed each level of Chester step test, pre- and post- dancing intervention (total n = 21).

	Number of Participants Completing Stage				
	Level 1	Level 2	Level 3	Level 4	Level 5
Pre-Intervention	21	21	16	8	1
Post-Intervention	21	21	21	13	3

**Table 4:** Heart rate (HR) recorded during the final seconds of Level 1, 2 and 3, and Mean HR calculated from averaged individual HR values recorded during the final seconds of Level 1, 2 and 3 of the Chester Step test conducted pre- and post-dancing intervention (n = 21). Values are mean ± SD.

	Pre	Post	Difference	95% CI	*p*-Value
Level 1	102 ± 13	98 ± 12	−4.3	−0.69, 9.26	0.088
Level 2	112 ± 13	106 ± 12.6	−5.3	1.84, 8.83	0.005
Level 3	122 ± 14	115 ± 14	−6.9	2.28, 11,59	0.006
Mean	111 ± 15	106 ± 14	−5.4	2.99, 7.80	0.000

CI, confidence interval for the difference between means.
